# Testing central auditory processing abilities in older adults with and without dementia using the consonant-vowel dichotic listening task

**DOI:** 10.3389/frdem.2023.1207546

**Published:** 2023-11-17

**Authors:** Jenna Littlejohn, Daniel J. Blackburn, Annalena Venneri

**Affiliations:** ^1^Department of Neuroscience, University of Sheffield, Sheffield, United Kingdom; ^2^Manchester Centre for Audiology and Deafness, University of Manchester, Manchester, United Kingdom; ^3^Department of Life Sciences, Brunel University London, London, United Kingdom; ^4^Department of Medicine and Surgery, University of Parma, Parma, Italy

**Keywords:** dementia, mild cognitive impairment, dichotic listening, auditory laterality, memory assessment service

## Abstract

**Background:**

Hearing loss and dementia are linked, although the roles of peripheral and central auditory dysfunction are not well defined. Many behavioral measures of hearing are confounded by the overlapping cognitive functions required to perform the tests.

**Objective:**

To collect pilot data to identify how central auditory function, measured using a dichotic listening test that indexes both auditory and cognitive components under different attentional conditions, differs among people with mild cognitive impairment (MCI), dementia and controls, and how performance relates to neuropsychological results.

**Method:**

Fifty-eight participants (17 MCI, 11 dementia and 30 controls) undertook hearing screening, the Bergen consonant-vowel dichotic listening paradigm, and a short battery of neuropsychological tests chosen to index attention and executive control. Dichotic listening was assessed under three attentional conditions (non-forced, forced right ear and forced left) amongst older adults with normal cognitive function, MCI and dementia.

**Results:**

We report two main findings: (a) The expected right ear advantage under non-forced conditions, was seen in controls and patients with dementia but not in people with MCI, who showed equal numbers of correct responses from both ears (i.e., a lack of asymmetry); (b) Performance under forced attentional conditions was significantly associated with disease progression (i.e., control > MCI > dementia) and performance on the cognitive tasks.

**Conclusion:**

The reduction in asymmetry on dichotic listening tasks may be a marker of MCI and reflect underlying compensatory mechanisms. Use of this test could aid stratification of patients with memory disorders. Whether abnormalities could predict dementia onset needs longitudinal investigation in a larger sample.

## Introduction

Hearing loss (HL) at midlife has been included as one of the potentially modifiable risk factors for the prevention of dementia (Livingston et al., 2020), with the largest population attributable fraction. The mechanism of this relationship is still not well understood due to overlapping symptoms, complexities with testing and confounding factors (Littlejohn et al., 2020; Brewster et al., 2022). Although pure tone audiometry is still the gold standard clinical assessment for HL, hearing difficulties are not restricted to the peripheral auditory system, as patients with dementia often exhibit poor performance on tasks of central auditory processing (Grady et al., 1989; Gates et al., 1995; Strouse et al., 1995). However, there remains a limited understanding of central auditory dysfunction in dementia. Patients with Alzheimer's disease (AD), the most common cause of dementia, perform significantly worse on auditory processing tests including measures of sound localization, speech discrimination and timbre discrimination when compared with matched controls (Strouse et al., 1995; Iliadou and Kaprinis, 2003), and these deficits may precede the onset of dementia (Gates et al., 2002). For some auditory processing measures, there appears to be a stepwise performance according to clinical disease progression (Idrizbegovic et al., 2011), but other studies have reported no associations with dementia severity or duration of dementia (Kurylo et al., 1993; Krishnamurti et al., 2011).

As intact auditory and cognitive functions are required for completing these behavioral tasks of central auditory function, lower performance could be due to disruption in language comprehension (Blair et al., 2007), temporal lobe atrophy and inhibition of attention processes (Grady et al., 1989), i.e., poorer performance due to the cognitive load of the task. However, AD pathology has been found along the auditory processing pathway (Ohm and Braak, 1989), and a specific pattern of distribution of plaques and neurofibrillary tangles has been reported throughout the auditory nuclei, the primary auditory and auditory association cortices (Sinha et al., 1993). This evidence may suggest that underlying pathology, at least in part, is contributing to the impairment on peripheral hearing and auditory processing tasks. Therefore, understanding central auditory function and dysregulation may be an opportunity to identify people at risk of AD and eventually dementia (Gates et al., 2002).

Due to the range of measures and vast differences in methods used to investigate cognitive and auditory function, there is no agreed gold standard test for measuring central auditory function in people with dementia. As top-down cognitive processes are involved in the processing of speech (Eysenck and Keane, 2005), using a paradigm that can separately index auditory and cognitive function may help to understand further the link between HL and cognitive decline in aging and pathology. A non-invasive technique to investigate central auditory processing is the consonant-vowel (CV) forced-attention dichotic listening paradigm (Hugdahl and Andersson, 1986; Hugdahl, 1995). This can be used to investigate both bottom-up (auditory) and top-down (cognitive) influences, by simultaneous presentation of different CV syllable pairs to each ear (Broadbent, 1954; McCullagh, 2013). There are two attentional conditions: non-forced attention and forced-attention tasks. During the non-forced task, participants tend to report more correct responses from the right ear relative to the left, demonstrating a right-ear advantage (REA) (Hugdahl and Andersson, 1986; Hugdahl et al., 2001; Foundas et al., 2006; Zenker et al., 2007; Takio et al., 2009; Saetrevik, 2012). This REA is a global phenomenon (Bless et al., 2013) that reflects the anatomical arrangement of the auditory system, with stronger contralateral pathways to the left hemisphere dominant for linguistic processing (Kimura, 1967). This results in slower and less accurate processing from the left ear (Asbjornsen and Hugdahl, 1995; Langers et al., 2005), that is delayed as input must first travel across the corpus callosum (Hugdahl et al., 1999). Under the forced-attention conditions, participants are instructed to attend to either the right or left ear. Healthy participants are able to modulate their attention to increase or decrease the bottom-up driven REA. The degree to which the REA can be modulated is dependent on many factors and individual differences including age, peripheral hearing abilities and cognitive function (Takio et al., 2009). Older adults often have stronger REA and a reduction in inhibition of the REA under the forced-left condition (Hugdahl et al., 2001; Takio et al., 2009). This effective management of competing signals involves cognitive processes such as short-term memory, shifting attention and competitive inhibition (Gates et al., 2008). Furthermore, the neural processing demands differ under the forced conditions, where there is a stronger activation of the prefrontal cortex and caudate nucleus under the forced-left ear task compared with non-forced- and forced- right ear conditions (Kompus et al., 2012), suggesting the involvement of different cognitive processes. Thus, the CV dichotic listening paradigm involves examination of three different auditory-cognitive processes: (1) lateralized perceptual processing under the non-forced condition, (2) attention under the forced-right condition, and (3) executive cognitive control during the forced-left condition (Hugdahl et al., 2009).

Other measures of dichotic listening, such as the dichotic digits or dichotic sentence identification test, have shown an association between performance and risk of AD (Gates et al., 2008; Mohammed et al., 2022). These measures, however, take longer to administer and may demand greater processing abilities due to linguistic complexity of the task (sentences), and therefore can be influenced by cognitive dysfunction (Gates et al., 2010; Bouma and Gootjes, 2011). The CV dichotic listening paradigm has not been used to investigate auditory-cognitive processing in people with dementia, nor have individual ear test scores been related to cognitive status or neuropsychological performance. This paradigm may be a useful measure of central auditory function in AD and neurodegenerative conditions, due to the semantically meaningless CV stimuli and the short and easy administration of the task.

The aim of this study was to collect feasibility and pilot data to measure how central auditory processing, measured using the CV dichotic listening paradigm is affected by neurodegenerative cognitive impairment. The objectives were three-fold: (1) to compare performance and laterality index under non-forced conditions; (2) to compare performance on the forced-attentional paradigms, overall, and in relation to individual ear gain scores for the forced-right and forced-left conditions; and (3) to investigate how overall task performance is associated with cognitive scores.

## Methods

### Participants

A total of 65 participants were recruited to this study. Of these, 35 participants with neurodegenerative cognitive impairment were recruited from the memory and dementia clinic at the Royal Hallamshire Hospital, Sheffield, UK. Inclusion criteria were mild to moderate typical cases of neurodegenerative cognitive impairment, including both patients with a diagnosis of MCI and those with dementia. Exclusion criteria were cognitive impairment with suspected functional, vascular or secondary etiology and MMSE scores below 15. Four participants were subsequently excluded based on their diagnosis, and a further three were excluded as they could not complete the dichotic listening task, leaving a total of 28 participants for subsequent analysis.

These participants were further categorized into MCI or dementia in order to investigate any differences according to disease severity. There were 17 with amnestic MCI and 11 with dementia. The dementia group included diagnoses of AD (*n* = 5), dementia with Lewy bodies (*n* = 2), frontotemporal dementia (*n* = 3), and corticobasal degeneration (*n* = 1). Patients were diagnosed based on multidisciplinary evidence and according to standard clinical criteria for AD (McKhann et al., 2011), dementia with Lewy bodies (McKeith et al., 2005), frontotemporal dementia (Englund et al., 1994), and MCI (Albert et al., 2011).

The patient group were compared with 30 age-, sex-, and education- matched controls, recruited as volunteers through various local advertisements and through word of mouth.

Ethical approval was obtained from the University of Sheffield Medical School (Ref: 002853) and NRES Committee North East- Newcastle and North Tyneside (Ref: 170445, 15/NE/0152). All participants gave their written informed consent.

### Audiometric screening

All participants undertook basic audiometric screening using a CE70 Handheld Pure Tone Warbler in a quiet clinical room to evaluate peripheral hearing levels for the purpose of ruling out moderate to profound cases and try to ensure no participants had interaural threshold differences at any of the frequencies. The modulation level was ±10%, and dB hearing levels ranged from 20 to 70. The tones were presented in sound field, directed at each ear separately, and a Bilsom^®^ 303L ear bud was used to occlude the non-test ear (Kramer, 2014). The tones were presented for a duration of between 1 and 3 s, with a stimulus onset asynchrony varying between 1 and 4 s to ensure the participant could not predict the presentation of the next tone (British Society of Audiology, 2018). The average of frequency specific responses to the stimuli at 500, 1,000, 2,000, and 4,000 Hz are reported in Table 1 and separately for each participant and ear in Table A1.

**Table 1:** Descriptive demographic statistics for each group.

**Group**	**Age (years)**	**Education (years)**	**Gender (M/F)**	**MMSE**	**Hearing threshold average (dB)**	***N***
Control	68.53 (10.43)	12.83 (2.47)	17/13	28.90 (1.06)	25.75 (7.51)	30
MCI	66.12 (10.83)	12.00 (2.26)	13/4	25.59 (3.26)	32.72 (14.95)	17
Dementia	68.18 (7.67)	11.64 (2.73)	5/6	23.45 (5.70)	26.36 (8.87)	11

Data in brackets represent the standard deviation.

MMSE, Mini Mental State Examination, which scores can range between 0 and 30; N, number of participants.

### Dichotic listening task

The mobile device app version of the Bergen CV dichotic listening test, iDichotic (Bless et al., 2015), was presented on an iPad tablet via a set of JVC circumaural headphones. This app version has high reliability and validity when compared with lab based tests (Bless et al., 2015). The auditory stimuli consisted of six consonant-vowel sounds (-ba, -da, -ga, -pa, -ta, and -ka), a pair of which were randomly assigned and simultaneously presented to the right and left ears, using all 36 possible combinations. Before the trials started, the minimum hearing levels for each ear were designated by the participant using a horizontal volume scroll bar, where a 1,000 Hz tone was adjusted to the point it became “just inaudible”. This helped to ensure the volume was appropriate for each participant and to adjust for any discrepancy in interaural hearing thresholds.

The task consisted of three conditions: the non-forced (NF) attention paradigm, the forced right (FR) attention paradigm, and the forced left (FL) attention paradigm. The NF is a non-directional condition where the participant was asked to report the most dominant sound (or the sound that was heard best) from either ear. Correct responses that matched stimuli played in either ear were recorded as correct and reported separately according to each ear. During the two forced listening paradigms, participants were directed to attend selectively to the right (FR) or the left (FL) ear and report only the syllable heard in this ear, ignoring the stimulus in the contralateral ear. For each condition, consonant-vowel pairs were presented at random for 30 trials, with the NF condition first followed by the FR or FL that was chosen at random by the app. For each participant and condition, the number of correctly reported syllables were recorded for each ear. Correct results from the FL and FR conditions were summed to give an overall score, and a laterality index (LI) was obtained to indicate the percentage difference between correct left ear and right ear responses for each condition.

### Cognitive testing

All participants undertook a short battery of cognitive tests, chosen to index closely the cognitive functions involved with the dichotic listening paradigm. These included the Mini-Mental State Examination (MMSE) (Folstein et al., 1975), as a global measure of cognitive function, the digit cancellation (Spinnler and Tognoni, 1987) to index attention, and the category fluency task (semantic fluency) and letter fluency task (phonemic fluency) (Lezak, 2004) to measure executive control.

### Analyses

All statistical analyses were undertaken using IBM SPSS Statistics for Windows, version 24. A series of one-way ANOVAs was carried out to investigate differences in demographic characteristics, LI and dichotic performance between the three groups. LI was computed using the following equation: (Correct RE – correct LE)/(Correct RE + Correct LE) × 100. The LI can range from −100 to 100, with negative values representing a left ear advantage and positive values representing a REA. Measures for overall scores were made up of FR+ FL ear performance, and for each forced listening task alone, gain scores were computed to demonstrate the increase in responses to the directed ear with respect to the NF condition, e.g., FL(L)-NF(L) and FR(R)-NF(R).

For all analyses, level of significance was set to α = 0.05 and effect sizes were provided as measures of explained variance (η^2^). *Post-hoc* comparisons were conducted using Tukey HSD test.

Associations between neuropsychological testing and overall dichotic listening scores for the whole group were carried out using the non-parametric Spearman's rho correlation coefficient, with corrected *p*-values at *p* = 0.0125 to control for multiple comparisons between the 4 tests. Gain scores computed as above, were used to test the relationship between performance on FR and FL tasks and neuropsychological performance.

## Results

### Demographic characteristics

The demographic characteristics of all groups are reported in Table 1. There were no statistically significant differences in age (*p* = 0.726), years of education (*p* = 0.305), gender (*p* = 0.228), or peripheral hearing levels (*p* = 0.086) between the three groups, but as expected, MMSE scores were significantly different between groups [*F*_(2, 55)_ = 14.587, *p* < 0.001, η^2^ = 0.347]. Tukey *post-hoc* test revealed that MMSE scores were statistically significantly lower for the MCI patients (25.59 ± 3.26 points, *p* = 0.002) and dementia patients (23.45 ± 5.70 points, *p* < 0.001) compared with controls (28.90 ± 1.06 points). There was no statistically significant difference between MCI and dementia groups (*p* = 0.186).

### Non-forced condition—Lateralized perceptual processing

**Figure 1:**
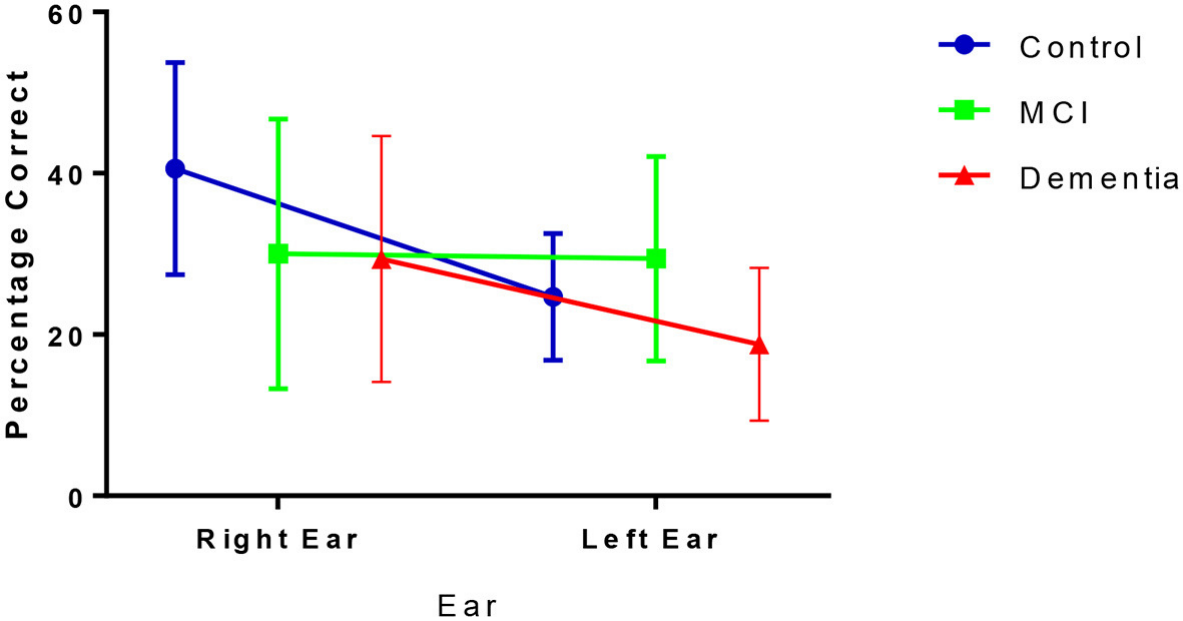
Correct (mean and SD) responses from right and left ear inputs under the NF condition.

During the NF condition, participants' percentage correct scores for each ear varied across the groups (Figure 1). Figure 1 shows that, although higher in the control group, both the control group and dementia group reported more correct answers from the right ear relative to the left as demonstrated by a positive laterality index of 24.4% (SD = 24.69) for controls and 17.19% (SD = 38.05) for dementia patients. Conversely, patients with MCI showed no preference for ear advantage laterality and reported, on average, similar numbers of correct responses from both ears (LI = −2.65%, SD = 41.55).

There was a significant difference between NF LI amongst the three groups [*F*_(2, 55)_ = 3.680, *p* = 0.032, η^2^ = 0.12], driven by the different scores between the MCI and controls (*p* = 0.024) rather than controls and dementia (*p* = 0.810) or MCI and dementia (*p* = 0.274; Table 2).

**Table 2:** Laterality index (LI) for all groups across the 3 tasks.

**Group**	**Non-forced (NF)**	**Forced right (FR)**	**Forced left (FL)**
Control	24.4 (24.69)	28.03 (30.03)	1.90 (38.33)
MCI	−2.65 (41.55)	9.06 (41.26)	−2.23 (34.56)
Dementia	17.19 (38.05)	24.21 (38.33)	13.19 (38.40)

Data in brackets represent the standard deviation. Laterality index ranges from −100 to 100, with negative values representing left ear advantage and positive values representing a right ear advantage.

### Forced conditions

**Figure 2:**
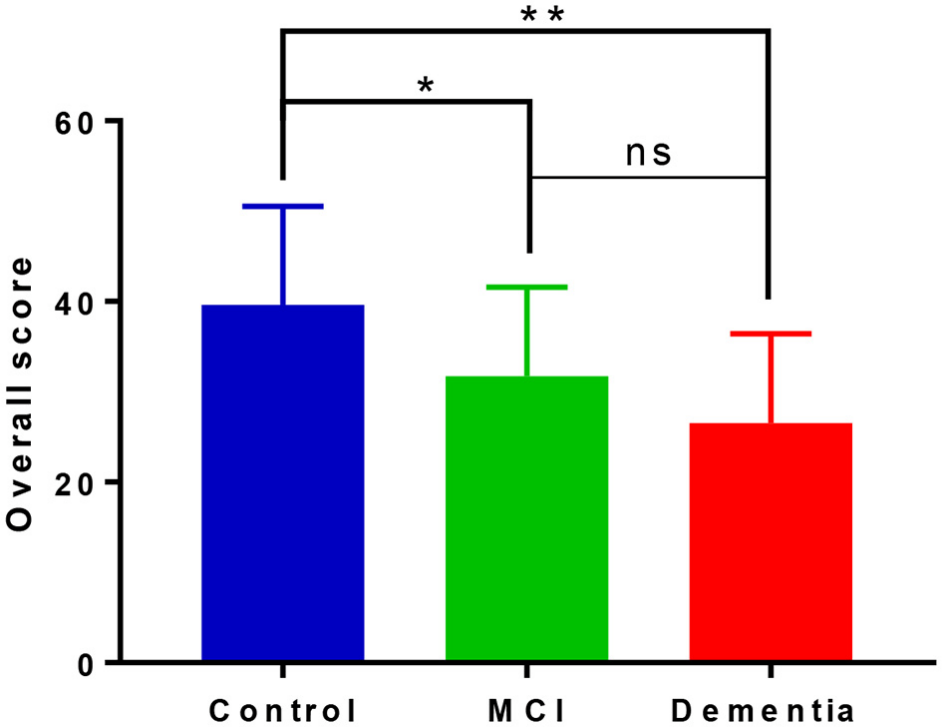
Overall performance on forced listening tasks on the iDichotic task. Overall scores include performance on forced right and forced left listening tasks. NB, Error bars denote standard deviation; *p < 0.05, **p < 0.01, ns, not significant.

There appeared a stepwise performance in overall scores on the iDichotic as shown in Figure 2, and significant mean difference between the groups [*F*_(2, 55)_ = 9.672, *p* < 0.001, η^2^ = 0.260]. Performance was significantly lower for MCI patients with respect to controls (mean difference of 8.83, *p* = 0.013) and dementia patients compared with controls (mean difference of 13.99, *p* < 0.001), with a non-significant lower performance for dementia patients relative to the MCI group (mean difference of 5.16, *p* = 0.373).

### Forced-right—Attention

**Figure 3:**
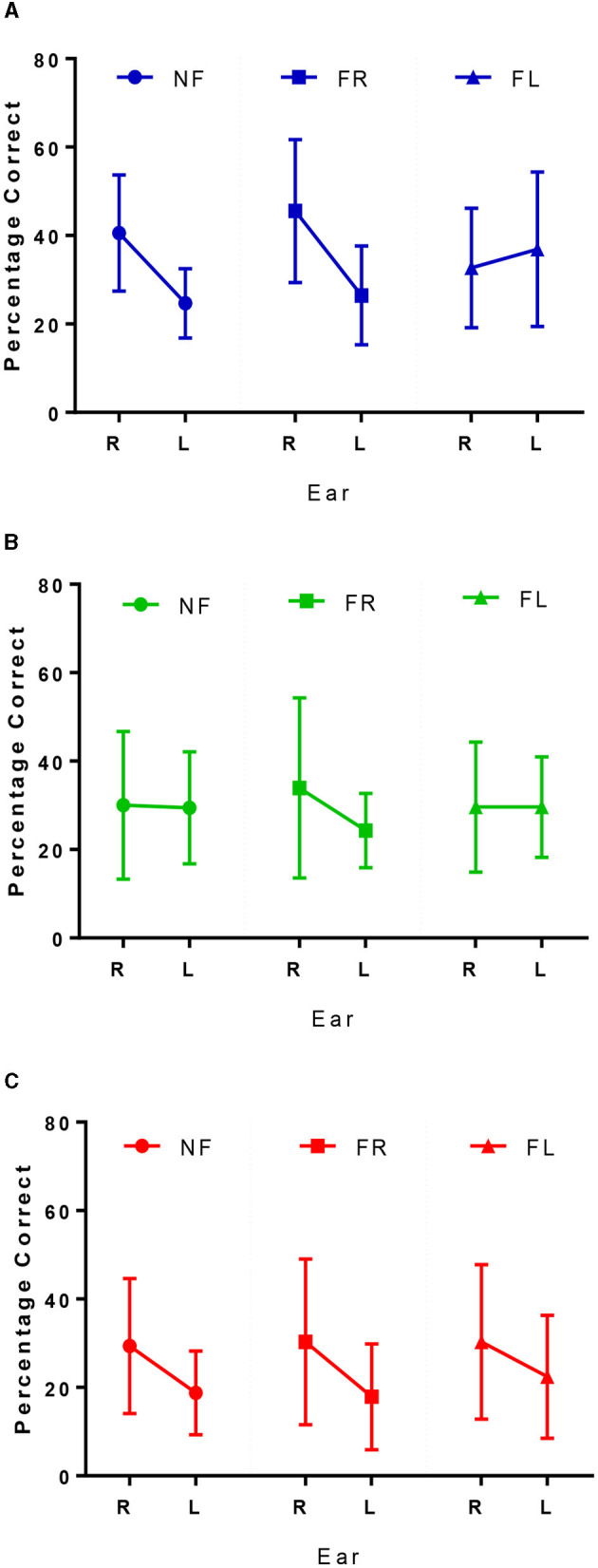
Correct right and left ear responses for controls in blue **(A)**, MCI patients in green **(B)**, and dementia patients in red **(C)** across the 3 conditions. NF, non-forced task; FR, forced right task; FL, forced left task.

Overall, there was a positive shift in LI to the right under the FR condition; although comparisons of LI scores from NF vs. FR conditions across the three groups demonstrated no significant difference in LI change [*F*_(2, 55)_ = 0.897, *p* = 0.414]. Controls could, on average, increase their correct responses from the right ear relative to the NF condition (Figure 3A). Similarly, as shown in Figure 3B, MCI patients increased their responses from the right ear and decreased the number of correct responses from left ear relative to the NF condition, to achieve a shift toward right laterality. Finally, Figure 3C shows that dementia patients could marginally (non-significant mean difference of 0.90%) increase their correct responses to the right ear, but still showed a right LI similar to the NF condition.

### Forced left—Executive cognitive control

Overall, there was a shift in LI to the left under the FL condition as shown in the final column of Figure 3. Figure 3A shows controls were able to overcome their REA by reducing the number of responses from the right ear and increasing the number of correct responses from the left. MCI patients could not increase their responses to the left ear relative to the NF condition, and patients with dementia still reported more correct responses from the right ear, although there was an increase in left ear responses relative to the NF condition (Figure 3C). Results from the repeated measures ANOVA showed there was a significant difference between the ability to attend to the left, relative to the NF condition between the three groups [*F*_(2, 55)_ = 3.89, *p* = 0.026, η^2^ = 0.125]. Tukey *post-hoc* comparisons indicated that this difference was only significantly different between controls and patients with MCI (*p* = 0.036) but not between controls and dementia patients (*p* = 0.230) or MCI and dementia patients (*p* = 1.00).

The change in LI for FL relative to NF task was independent of age (*r* = −0.124, *p* = 0.356), education (*r*_*S*_ = −0.219, *p* = 0.099) and peripheral hearing (*r*_*S*_ = 0.067, *p* = 0.620) across all participants.

### Dichotic listening and neuropsychology

There was no association between NF LI and cognitive performance on any of the tasks. The associations between the forced listening tasks for overall performance (FR+FL), and gain scores for the FR condition and FL condition, relative to the NF condition, with cognitive test scores are shown in Table 3.

**Table 3:** Association between forced attention tasks and neuropsychology.

**Cognitive task**	**Overall**	**Forced right**	**Forced left**
MMSE	**335 (0.010)** ^*^	0.077 (0.564)	**0.464 (<0.001)** ^*^
Digit cancellation	**0.495 (0.001)** ^*^	0.149 (0.277)	**0.381 (0.004)** ^*^
Semantic fluency	**0.412 (0.002)** ^*^	0.55 (0.689)	**0.542 (<0.001)** ^*^
Phonemic fluency	0.256 (0.062)	0.030 (0.832)	0.222 (0.106)

^*^Correlation significant at p = 0.0125 level. The bold values are to demonstrate significance at the p = 0.0125 level as demonstrated by *.

Overall = Overall performance (FR+FL), Forced Right = Forced Right task, gain in right ear correct responses; Forced Left = Forced left task, gain in left ear correct responses.

On the whole group level, overall performance on the forced attention tasks showed a significant correlation with performance on the MMSE, Digit Cancellation and Semantic Fluency tasks. These significant correlations appear to be driven by the inhibition in attention to the right ear and increase in attention to the left ear under FL task conditions (Table 3).

## Discussion

Under NF conditions, controls reported more correct answers from their right ear, and fewer correct answers from their left ear, resulting in the expected REA. The laterality index and percentage correct right and left ear responses are consistent with those previously reported in the literature using this paradigm in the English language (Bless et al., 2013, 2015). Patients with dementia had a pattern of laterality that followed that of controls, however the number of correct answers from both the right and left side were substantially reduced (see Figure 2). Using the same iDichotic listening paradigm to investigate NF LI in a large sample of aging adults, Westerhausen et al., reported that the REA increases with age, and it is driven by a decline in left ear performance since responses from the right ear remained relatively constant (Westerhausen et al., 2015). Here, we report a different profile to that of “normal” aging in the dementia condition: a decline in the number of correct responses from both the right and left ears, that suggests that it might be due to reduced auditory processing as this NF task does not rely on higher order cognitive processing (Westerhausen et al., 2015).

Most interestingly, the pattern of responses was entirely different for the MCI patients. Under NF conditions, there was no asymmetry as participants reported equal numbers of correct answers from the right and left ears. In AD, levels of atrophy are more severe in the left hemisphere, and precede changes in the right hemisphere (Loewenstein et al., 1989; Janke et al., 2001; Thompson et al., 2003; Donix et al., 2013), and therefore in the MCI phase, a compensatory rearrangement mechanism may be occurring. The right hemisphere may be recruited to compensate for the underlying pathological changes developing in the preponderant left hemisphere, reflecting bilateral processing, and resulting in the lack of asymmetry.

Our results closely support findings from an fMRI study investigating auditory laterality in pre-manifest and manifest Huntington's disease (HD), using an auditory stimulation programme. The authors reported controls had a mainly left hemispheric activation, but this was unexpectedly reversed in people with pre-manifest HD to an increased right activation. The pre-manifest HD groups were further split by time to conversion, into close and far, that then led to the finding that the “far” patients had higher left hemispheric activation, but the “close” pre-manifest HD group showed no difference in activation between the right and left hemisphere (Saft et al., 2008). This mirrors the behavioral results in our MCI group, lending support to compensatory changes via recruitment of additional brain areas during neurodegenerative processes.

A recent study that used the same dichotic listening task in a dual-task paradigm with over ground walking lends further support to our findings of reduced asymmetry in MCI (Gorecka et al., 2021). Previous work had shown the attentional demands of the dual task paradigm to evoke asymmetric gait effects on healthy controls, with the aim of this study to investigate any differences in a group of people with amnestic MCI. As the authors did not assess dichotic listening as a single task, we cannot directly compare performance between the studies, but the authors did report a distinct lack of gait asymmetries in MCI patients compared with the matched controls. They concluded attentional demands affect performance between controls and MCI patients in very different ways, and taken together, this information supports the notion that attentional demands may reduce the asymmetry to prioritize task performance, as we have seen in the present study.

It is unclear why adaptive processes may be more active for these individuals, but it may signify a differing etiology or stage of neuropathological disease progression. Various fMRI studies have reported altered activity and interaction between auditory and higher order cortices in patients with MCI and AD when compared with controls (Dhanjal et al., 2013), and greater hippocampal activity in response to memory tasks in MCI that is not seen in AD (Dickerson et al., 2005), findings that could be interpreted as reflecting a compensatory effort to sustain performance. A recent study using a binaural integration paradigm, investigated detection and storage of binaural temporal fine structure of wideband noise amongst 4 groups of healthy controls, people with subjective cognitive problems, amnestic MCI and AD (Wang et al., 2022). The authors report that phase synchrony of P_2_ wave (a late auditory processing stage) appeared as a “U shaped curve” when comparing the groups; initially declining from controls to subjective cognitive problems, to MCI, but then increasing again from MCI to AD. They suggested this is possibly reflective of a neural adaptation mechanism or indicative of systemic degradation (Wang et al., 2022). These findings are in line with our own results, demonstrating a different pattern of performance in MCI participants with respect to controls and dementia patients.

Under the forced conditions, as expected, we report a linear reduction in the overall dichotic listening scores relating to the severity of cognitive impairment. On average, controls performed better than MCI patients, who performed better than dementia patients (Figure 2). This declining performance amongst the groups has been reported previously in the literature (Idrizbegovic et al., 2011) and helps to corroborate this idea of compensatory mechanisms occurring in MCI to increase task performance that are then lost or ineffective in supporting performance at the dementia phase.

As expected, at the group level there was a (non-significant) positive shift in LI to the right under the FR task. Further investigation into ear performance demonstrated that, to varying degrees, all three groups could increase their responses from the right ear relative to the NF condition (Figures 3A–C). This is in keeping with the literature, due to the perceptual salience of the right ear stimulus, even in people with dementia (Bouma and Gootjes, 2011). Under the FL conditions there also was an overall shift in LI to the left that was the strongest for controls (Table 2) and almost non-apparent in the MCI group, reflecting a significant difference in the ability to attend to the left side that was independent of age, education, and hearing levels. Figures 3B, C shows the little change from NF/FR conditions in the MCI and dementia patients. Due to the absent REA under NF conditions in the MCI group, it is unclear what may be happening, but within the dementia group there is a bottom-up REA that cannot be modified by top down processing (Idrizbegovic et al., 2011).

Associations between dichotic listening performance and neuropsychological testing outlined in Table 3 further substantiates our support for use of the CV paradigm in separately indexing auditory and cognitive function (Hugdahl et al., 2009). We report no association between NF LI and cognitive performance maintaining the notion that NF conditions index bottom-up auditory processing that is unrelated to higher-order cognitive function but demonstrate differences in integrity of the central auditory system between healthy controls, those with MCI and dementia. The forced listening components of the test that index cognitive function are associated with disease progression (Figure 2) and neuropsychological performance (Table 3). We report significant correlations at the group level for overall scores and the FL task, but due to the small sample size, the small and non-significant associations between cognitive tests and the FR condition, does not preclude the possibility that there might be further effects that our study was not sufficiently powered to detect.

A recent study found that interaural differences in sensory processing may affect performance on dichotic listening tasks (Ianiszewski et al., 2021). We reported non-significantly slightly higher peripheral hearing in patients with MCI with respect to controls (Table 1), and although our results (Table A1) suggest no major interaural differences in hearing abilities, we cannot exclude the possibility that peripheral hearing levels may have impacted dichotic listening performance and thus could be confounding the results. However, other authors have also reported no difference in peripheral auditory function between patients and age-matched controls (Gates et al., 1995). Due to the equipment used in this study, it was not possible to report accurate hearing thresholds and future studies should use equipment capable of determining interaural differences in hearing abilities with a higher level of precision. However, in this study for the dichotic listening experiment, participants were able to alter sound levels to be the most comfortable for them in each ear independently, enabling participants to overcome any minor subjective differences in interaural hearing abilities. Again, we acknowledge that this was only for one mid-range frequency and there could be differences in higher frequencies. Obtaining accurate hearing thresholds using standardized clinical audiometry would allow for further analysis into hearing thresholds and performance on the dichotic listening task. Furthermore, to be completely certain there is no effect of hearing ability on performance, it would be beneficial to compare the exact output levels for each ear and trial, but this was beyond the detection abilities of the app used in this study.

Another limitation of the present study is the small sample size and the heterogeneous etiology of impairment in the disease groups. As clinical assessment alone may be an insufficiently accurate predictor of disease pathology, a study with greater number of patients with similar etiology, e.g., by including CSF biomarker evidence for presence of AD would be recommended. Although the iDichotic app has high reliability [*r* (ICC) = 0.78] and validity [*r* (ICC) = 0.76–0.82] when compared with lab based measures (Bless et al., 2013), it would also be extremely useful to increase the number of trials for each participant and to follow the participants up over time to monitor repeat performance on this task so that LI could be indexed longitudinally, and consider how laterality on this task may change over time as reported in Saft et al. (2008). This would clarify if the performance were reflective of an overall generic impairment or if this pattern of performance is more reflective of underlying AD pathology into specific brain regions and their associated function. It is likely that repeated testing would overcome the limitation of sensitivity of audiometry testing and might allow this technique to be developed as a non-invasive test to track progression of disease in people with MCI or other prodromal cognitive state. A larger sample size with repeat testing would also allow to investigate interaction effects between ear, group, and condition.

To summarize, using a tool that can separate out bottom- up and top-down contributions to auditory processing, we report preliminary findings that lateralized perceptual processing performance is different for patients with MCI and patients with dementia when compared with a group of age, sex and education matched controls. We suggest this disruption of auditory processing under the NF condition may indicate compensatory mechanisms are occurring during neurodegenerative disease, reflecting bilateral processing rather than the usual left hemispheric dominance. It is possible that a task indexing auditory processing may be a more sensitive measure to detect subtle changes in cognitive and underlying neuronal function (Gates et al., 2002), thus subclinical deficits may manifest earlier than in neuropsychological testing. As there are no ceiling or practice effects, it is possible to investigate changes in cognitive control over time. Further investigation into the longitudinal effects of neurodegenerative processes on this task could result in this paradigm being used to aid stratification of patients with memory disorders, at risk of developing dementia. Identifying people who are at risk of dementia early is of benefit for access to treatments and interventions, or to implement preventative strategies, and has the potential for great public health implications in terms of costs to society.

## Data availability statement

The raw data supporting the conclusions of this article will be made available by the authors, without undue reservation.

## Ethics statement

The studies involving humans were approved by University of Sheffield Medical School (Ref: 002853) and NRES Committee North East-Newcastle and North Tyneside (Ref: 170445, 15/NE/0152). The studies were conducted in accordance with the local legislation and institutional requirements. The participants provided their written informed consent to participate in this study.

## Author contributions

JL, DB, and AV contributed to the design of the research. Implementation of the research and analysis of the results was undertaken by JL. JL wrote the first draft of the manuscript and all authors contributed to the final version. All authors contributed to the article and approved the submitted version.

## Appendix

**Table A1:** Audiometric screening results for each participant across four frequencies for right and left ears.

**Participant**	**500 Hz**		**1,000 Hz**		**2,000 Hz**		**4,000 Hz**	
	***R***	***L***	***R***	***L***	***R***	***L***	***R***	***L***
1	30	25	25	25	35	40	60	55
2	20	20	20	20	20	20	25	20
3	40	35	40	35	50	45	60	60
4	60	55	45	40	30	35	30	35
5	20	20	20	20	20	20	20	20
6	20	20	20	20	35	35	60	55
7	20	20	20	20	20	25	30	30
8	20	20	20	20	20	20	20	20
9	20	20	20	20	20	20	20	20
10	20	20	25	25	40	35	60	55
11	20	20	20	20	20	20	20	20
12	25	20	20	20	20	20	20	20
13	20	20	20	20	20	20	35	40
14	25	25	35	30	50	55	60	65
15	20	30	20	35	25	40	35	65
16	20	20	20	20	20	20	20	20
17	20	20	20	20	20	20	20	20
18	20	20	25	30	50	50	55	60
19	20	20	20	20	40	60	65	-
20	20	20	20	20	20	30	20	30
21	20	20	20	25	20	20	20	20
22	20	20	20	20	20	20	40	50
23	20	20	30	30	45	50	65	55
24	20	20	20	20	20	20	20	20
25	20	20	20	20	20	20	30	35
26	20	20	20	20	20	20	20	20
27	20	20	20	20	20	20	20	20
28	20	20	20	20	20	20	25	25
29	20	20	20	20	35	35	60	60
30	20	20	20	20	20	20	35	30
31	20	20	20	20	20	20	20	20
32	70	60	60	55	55	50	60	50
33	20	20	20	20	20	30	30	40
34	20	20	20	20	20	20	45	35
35	25	20	30	25	60	65	60	-
36	20	20	20	20	20	20	40	35
37	25	25	20	20	55	45	55	65
38	50	55	65	65	55	55	-	-
39	60	50	70	60	70	70	-	-
40	20	20	20	20	20	20	20	20
41	20	20	20	30	20	30	55	65
42	20	20	20	20	20	20	20	20
43	20	20	35	30	45	45	65	60
44	20	20	20	20	20	20	20	20
45	20	20	20	20	20	20	20	20
46	20	20	20	20	20	20	50	50
47	20	20	25	35	30	35	30	35
48	20	20	20	20	20	20	30	25
49	20	20	20	20	20	20	40	35
50	20	20	20	20	20	20	20	20
51	20	20	20	20	20	20	20	35
52	20	20	25	20	25	25	20	30
53	30	40	35	45	40	40	55	55
54	50	60	40	50	40	55	55	55

R, right ear; L, left ear.

NB, Missing data for individual ear scores for n = 4 participants.
